# From plastic bottle waste to functional adsorbent: a nanostructured bimetallic Al/Cd-BDC MOF for enhanced methylene blue removal

**DOI:** 10.1038/s41598-026-48178-y

**Published:** 2026-04-21

**Authors:** Mayas Saad, Abdelkarim Elhamad, Mohammad Deeb, Hussam Alrakkad

**Affiliations:** Department of chemistry, Faculty of science, Latakia University, Lattakia, Syrian Arab Republic

**Keywords:** Metal-organic framework (MOF), Bimetallic MOF, PET waste upcycling, Hierarchical nanostructure, Methylene blue, Adsorption, Chemistry, Environmental sciences, Materials science, Nanoscience and technology

## Abstract

The dual environmental crises of industrial water pollution and plastic waste accumulation necessitate the development of sustainable, high-performance materials derived from waste streams. In this work, a novel bimetallic metal-organic framework (MOF), designated as Al/Cd-BDC, was successfully synthesized through an innovative “waste-to-value” strategy, using terephthalic acid (TPA) derived from the chemical upcycling of post-consumer PET plastic waste. Morphological investigation unveiled spherical microstructures composed of needle-shaped nanocrystals, creating an urchin-like hierarchical architecture. This structure provides substantial porosity and a high specific surface area of 650.8 m^2^g^–1^, which enhances mass transport and creates highly accessible active sites. The synthesized Al/Cd-BDC demonstrated exceptional and rapid performance in the removal of methylene blue (MB) from aqueous solutions. Under optimized conditions (pH: 7; adsorbent dose: 0.6 g L^–1^; t: 30min), it achieved a removal efficiency of 98.97%, with a theoretical maximum adsorption capacity (Q_m, cal._) of 352.2 mg g^–1^. This superior performance is attributed to the synergistic effects of the bimetallic centers and the hierarchical nanostructure. Furthermore, the MOF exhibited excellent stability and reusability, maintaining over 97% removal efficiency after five consecutive cycles. These findings underscore the potential of Al/Cd-BDC as a robust and sustainable adsorbent for industrial wastewater treatment.

## Introduction

The confluence of accelerating industrial development and global population growth has precipitated two critical environmental challenges: the pervasive contamination of water resources and the overwhelming accumulation of plastic waste^1,2^. Industrial effluents, particularly from the textile, pigment, and paper industries, frequently discharge large volumes of wastewater containing synthetic organic dyes^3,4^. These dyes are often characterized by complex aromatic structures, rendering them resistant to biodegradation and conventional wastewater treatment methods^5,6^. Methylene blue (MB), a common cationic dye, is a frequently studied model pollutant due to its recognized toxicity and persistence in aquatic environments; prolonged exposure has been linked to significant health risks in humans^7,8^. Simultaneously, the burgeoning global production of plastics has led to a long-term pollution crisis, with polyethylene terephthalate (PET)-one of the most ubiquitous polymers in single-use packaging such as beverage bottles^9,10^-massively accumulating in landfills and natural ecosystems. The chemical inertness and low biodegradability of PET waste highlight an urgent need for innovative management solutions that move beyond simple disposal^11,12^. Addressing this dual predicament necessitates a paradigm shift towards integrated and sustainable technologies that can create value from waste streams while simultaneously tackling pollution, forming the core motivation for this research.

The principles of a circular economy offer a powerful framework for this challenge, promoting the transformation of waste into valuable chemical feedstocks^13,14^. PET, a polyester of terephthalic acid (TPA) and ethylene glycol, is an ideal candidate for chemical upcycling^10,15^. It has been robustly demonstrated that waste PET can be efficiently depolymerized via chemical methods, such as alkaline hydrolysis or glycolysis, to recover high-purity TPA^16–18^. This waste-derived TPA presents a sustainable, low-cost alternative to the petroleum-derived TPA used in industrial synthesis, thereby reducing dependence on fossil fuels and lowering the environmental burden of chemical manufacturing^13,19^.

Metal-organic frameworks (MOFs) have emerged as highly promising materials for environmental remediation, owing to their tunable porosity, exceptionally high surface areas, and diverse chemical functionalities that allow for precise molecular sieving and selective adsorption^20^. This “waste-to-value” strategy, particularly the upcycling of PET to terephthalic acid (TPA), is especially synergistic with the burgeoning field of MOFs as TPA serves as a fundamental organic linker in their construction^21^. These unique properties have established MOFs as superior materials for a vast range of applications, including gas storage and separation^22,23^, heterogeneous catalysis^24,25^, and chemical sensing^26^. In the context of environmental remediation, MOFs have shown exceptional promise as high-performance adsorbents for the removal of organic pollutants from water^27,28^. The engineering of MOFs with hierarchical nano-architectures is particularly advantageous, as the presence of interconnected micro- and mesopores can significantly enhance mass transport kinetics, allowing pollutant molecules to more readily access the internal active sites^29,30^. However, the performance of conventional monometallic MOFs can sometimes be limited by factors such as moderate structural stability, particularly in aqueous environments, or insufficient active sites for demanding applications^31^. To overcome these limitations, the synthesis of bimetallic or multivariate MOFs has emerged as a powerful strategy. This approach allows for the fine-tuning of electronic properties and the creation of synergistic effects between different metal centers, which can lead to enhanced structural stability and superior adsorptive or catalytic performance^32–34^. In this work, Al/Cd-BDC was rationally designed leveraging aluminum’s well-established aqueous stability with cadmium’s coordination versatility for cationic dye adsorption.

Herein, we report the successful synthesis of a novel, nanostructured bimetallic (Al/Cd-BDC) MOF using a "waste-to-value" approach. The essential terephthalic acid (TPA) linker was sustainably derived from the chemical upcycling of post-consumer PET plastic waste. The synthesized material was then comprehensively characterized and its performance was systematically evaluated for the removal of methylene blue (MB) from aqueous solutions. This work not only presents a high-performance adsorbent but also provides a compelling proof-of-concept for a circular economy model, transforming plastic waste into a valuable tool for environmental remediation.

## Experimental section

### Materials

Aluminum sulfate hexadecahydrate (Al_2_(SO_4_)_3_·16H_2_O, 98%), cadmium chloride monohydrate (CdCl_2_·H_2_O, 99%), N, N-dimethylformamide (DMF, ≥99.8%), sodium hydroxide (NaOH, ≥97%), hydrochloric acid (HCl, 37%), ethanol (C_2_H_5_OH, 99.8%), sodium chloride (NaCl, ≥99%), and methylene blue dye (MB) were purchased from Sigma-Aldrich (USA). All reagents were of analytical grade and used as received without further purification. Deionized water was used throughout the experiments. The organic linker, benzene-1,4-dicarboxylic acid (BDC), was synthesized from clear post-consumer PET water bottles collected locally, following our previously reported upcycling method^18^.

### Synthesis of MOF materials

The bimetallic Al/Cd-BDC MOF was synthesized via a solvothermal method. In a typical synthesis, the organic linker, terephthalic acid (BDC), was first dissolved in 25 mL of DMF to create the linker solution (0.5 g, 3.0 mmol). Separately, solutions of Al_2_(SO_4_)_3_·16H_2_O (0.95 g, 1.5 mmol) and CdCl_2_·H_2_O (0.61 g, 3.0 mmol) were prepared, each dissolved in a 5 mL DMF / 1 mL H₂O solvent mixture. The two metal solutions were combined and added to the BDC solution under stirring. The final mixture was sealed in a 50 mL Teflon-lined autoclave and heated at 120°C for 24 h. The resulting product was recovered by filtration, washed with DMF and ethanol, and dried at 120°C for 12 h. For comparative purposes, the monometallic analogues Al-BDC and Cd-BDC were synthesized under identical conditions using only the aluminum or cadmium precursor, respectively, while maintaining the same total metal-to-linker molar ratio.

### Characterization

The crystalline structure and phase purity of the synthesized materials were investigated using X-ray Diffraction (XRD). The patterns were recorded on a PHILIPS PW1730 diffractometer (The Netherlands) equipped with a Cu Kα radiation source (λ = 1.5406 Å). Fourier-Transform Infrared (FT-IR) spectroscopy was used to identify the functional groups of the organic linker and to confirm its coordination to the metal centers. The spectra were recorded in the 4000–400 cm⁻^1^ range on a PerkinElmer TWO spectrometer (USA), using the KBr pellet method. The morphology and elemental composition of the samples were investigated using a MIRA3 TESCAN scanning electron microscope (Czech Republic) equipped with an Energy-Dispersive X-ray Spectroscopy (EDS) detector. The images were acquired at an accelerating voltage of 20.0 kV. The specific surface area and porosity were determined by N_2_ adsorption–desorption isotherms at 77 K using a BELSORP-mini analyzer (Japan). Samples were degassed under vacuum at 120°C for 5 h prior to analysis. The specific surface area was calculated using the BET method, total pore volume from N_2_ adsorbed at P/P₀ ≈ 0.99, and pore size distribution from the adsorption branch using the BJH model. Thermogravimetric Analysis (TGA) was performed on a TA SDT Q600 instrument (USA). Samples were heated from 25°C to 600°C at a constant heating rate of 20°C/min under a continuous flow of inert argon gas to assess the thermal stability of the synthesized materials.

### Adsorption experiments

The adsorption performance of the synthesized Al/Cd-BDC MOF was evaluated for the removal of methylene blue (MB) from aqueous solutions. A stock solution of MB (1000 mg L^–1^) was prepared and diluted as needed. The optimization of adsorption parameters was carried out sequentially using a one-variable-at-a-time (OVAT) approach. All experiments were conducted at a controlled room temperature (25 ± 2 °C) with agitation provided by a magnetic stirrer (300 rpm).

First, the effect of contact time was studied to determine the equilibrium time. A series of experiments were conducted by mixing 20 mg of the adsorbent with 25 mL of a 10 mg L^–1^ MB solution at an initial pH of 7. Samples were collected at different time intervals ranging from 5 to 60min. The optimal contact time determined from this study was then used for all subsequent experiments. Second, using the optimal contact time, the effect of adsorbent dosage was examined. The mass of the adsorbent was varied from 5 to 30 mg (corresponding to 0.2–1.2 g L^–1^) in 25 mL of 10 mg L^–1^ MB solution at pH 7. The optimal dosage was selected for use in the following experiments. Third, the effect of initial solution pH was investigated over a range of 2–10. These experiments were conducted using the previously determined optimal contact time and adsorbent dosage. The initial pH of the solutions was adjusted using 0.1 M HCl or 0.1 M NaOH. Finally, to understand the effect of initial dye concentration and to generate the data for the adsorption isotherm study, the initial MB concentration was varied over the range of 10 – 200 mg L^–1^. These experiments were conducted under the optimal conditions of contact time, adsorbent dosage, and pH established in the preceding steps. Following each batch experiment, the adsorbent was separated from the solution by centrifugation (8000 rpm, 5 min). The residual MB concentration in the supernatant was analyzed using an Optima SP-3000plus UV-vis spectrophotometer (Japan) at its maximum absorbance wavelength (λ_max_ = 664 nm). All experiments were performed in triplicate, and the average values are reported. The adsorption capacity (q, mg g^–1^) and the removal efficiency (R, %) for all experiments were calculated using Eq. (1) and (2), respectively^35,36^.

1 $$q_{e} \, = \, \left( {C_{0} \, - \, C_{e} } \right) \times V \, / \, m$$

2 $$R \, \left( \% \right) \, = \, \left( {\left( {C_{0} \, - \, C_{e} } \right) \, / \, C_{0} } \right) \times 100$$

Where C_o_ and C_e_ (mg L^–1^) are the initial and equilibrium concentrations of MB, V (L) is the volume of the solution, and m (g) is the mass of the adsorbent.

## Results and discussion

### Characterization results

#### XRD

**Fig. 1:**
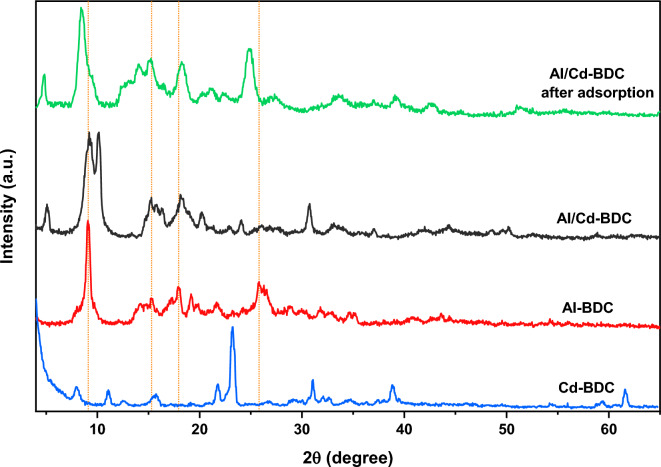
XRD patterns for Al-BDC, Cd-BDC, Al/Cd-BDC and Al/Cd-BDC after adsorption.

The Powder X-ray Diffraction (PXRD) patterns of the as-prepared materials are presented in Fig. 1. The pattern of the monometallic Al-BDC closely matches the experimental pattern reported for MIL-53(Al)^37^, confirming the successful synthesis of the MIL-53(Al) crystalline phase. Based on the crystallographic data from the Cambridge Crystallographic Data Centre (CCDC 220475), the main characteristic peaks observed at 2θ values of 9.1°, 15.3°, 18°, and 25.8° are indexed to the (101), (011), (202), and (411) crystal planes, respectively. The PXRD pattern of the bimetallic Al/Cd-BDC framework, closely resembles that of Al-BDC, indicating that the incorporation of Cd^2^⁺ ions results in minor structural modifications rather than a fundamental change of the parent framework. Crucially, a subtle but distinct anisotropic shift in the peak positions provides quantitative evidence for the homogeneous incorporation of Cd^2^⁺ ions into the MIL-53(Al) lattice. Specifically, the characteristic (101) peak for Al-BDC at 9.1° shifted to 9.3° in the Al/Cd-BDC sample, while the peak at 15.3° shifted to 15.2° and the peak at 18° shifted to 18.2°. This shift confirms the formation of a bimetallic solid solution. Finally, the PXRD pattern of the Al/Cd-BDC material after MB adsorption shows that the fundamental crystalline structure is well-preserved, confirming its high structural stability. However, subtle shifts in the positions of the main diffraction peaks are observed. This phenomenon, often referred to as a “breathing” effect or host-guest response, is characteristic of flexible MOFs like MIL-53 and is triggered by the interaction with guest molecules^38^. This provides compelling evidence that the MB molecules were adsorbed not only on the external surface but also within the framework’s pores, interacting directly with the crystalline lattice.

#### FTIR

**Fig. 2:**
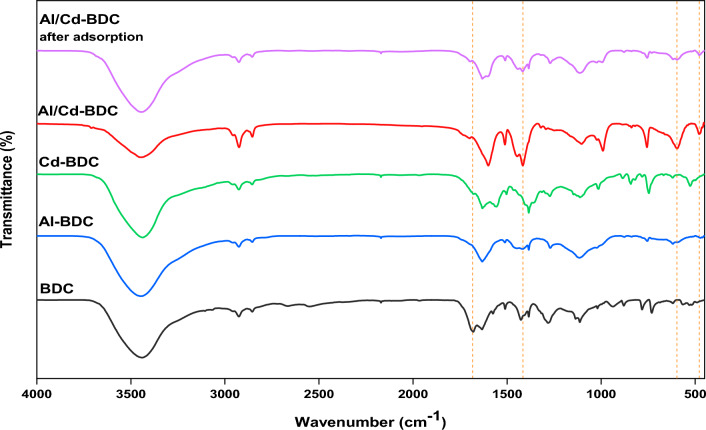
FTIR spectra for BDC, Al-BDC, Cd-BDC, Al/Cd-BDC and Al/Cd-BDC after adsorption.

The formation of the coordination framework and the binding mode of the terephthalic acid (BDC) linker were investigated using Fourier-transform infrared (FTIR) spectroscopy. The comparative spectra of the free BDC linker, monometallic Al-BDC, Cd-BDC and the bimetallic Al/Cd-BDC before and after adsorption are presented in Fig. 2. In the spectrum of the free BDC linker, a characteristic intense absorption band is observed at approximately 1685 cm^–1^, attributed to the C=O stretching vibration (ν(C=O)) of the carboxylic acid groups. Upon formation of the Al-BDC, Cd-BDC and Al/Cd-BDC frameworks, this band is significantly diminished and almost completely disappears. Concurrently, two new strong absorption bands emerge at approximately 1599 cm^–1^ and 1416 cm^–1^. These bands are assigned to the asymmetric (νasym (COO⁻)) and symmetric (νsym (COO⁻)) stretching vibrations of the deprotonated carboxylate groups, respectively^39^. The substantial reduction of the protonated carboxylic acid signal and the emergence of these prominent carboxylate modes provide definitive evidence that the BDC linker has been largely deprotonated and successfully coordinated to the metal centers. Furthermore, the appearance of new bands in the low-frequency region (480–600 cm^–1^) for the MOF samples, can be attributed to the formation of metal-oxygen (M-O) bonds^40^, further confirming successful coordination. The broad band centered around 3445 cm^–1^ in the MOF spectra is attributed to the O-H stretching vibrations of adsorbed and coordinated water molecules^40^, the presence of some residual intensity in the C=O region, coupled with this broad O-H band, may also suggest the presence of a small amount of unreacted BDC molecules, potentially trapped within the framework’s pores, which is consistent with the thermal analysis discussed in the TGA section. The structural integrity of the Al/Cd-BDC framework following the methylene blue (MB) adsorption process was also evaluated. The post-adsorption spectrum shows that the fundamental vibrational modes of the MOF are preserved, confirming its high structural stability. However, a subtle but distinct modification is observed in the main asymmetric carboxylate band. The band at 1599 cm^–1^ becomes broader and exhibits a new shoulder at a slightly higher wavenumber (~1630 cm^–1^). This change in band shape is attributed to the spectral overlap of the MOF’s carboxylate vibration with the characteristic aromatic C=C/C=N stretching vibrations of adsorbed MB molecules, which are known to have a strong absorption band in this region. This observation not only corroborates the stability of the framework but also serves as secondary evidence for the successful uptake of the dye.

#### SEM

**Fig. 3:**
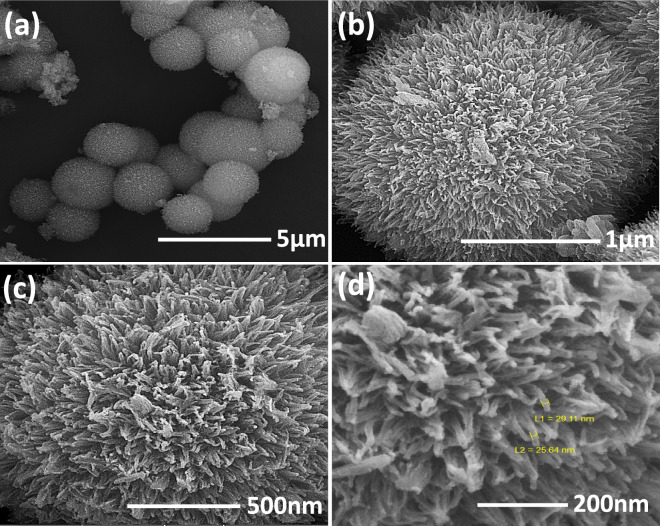
SEM images of the Al/Cd-BDC MOF.

**Fig. 4:**
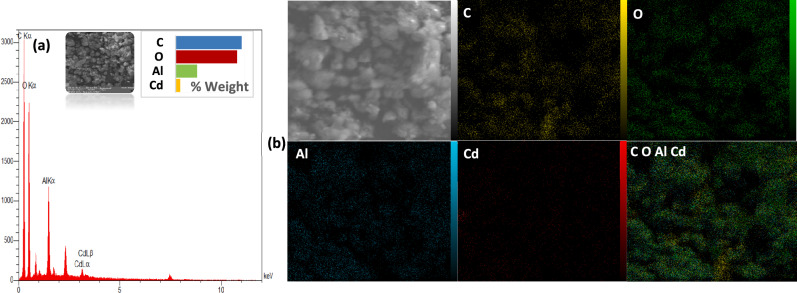
EDX spectrum of Al/Cd-BDC MOF(**a**), elemental mapping of Al/Cd-BDC MOF(**b**).

The morphology of the synthesized material was investigated by Scanning Electron Microscopy (SEM). The analysis revealed the formation of uniform, spherical microstructures with diameters ranging from 2 to 5 μm (Fig. 3a, b). At higher magnifications (Fig. 3c, d), it became evident that these microspheres possess a distinctive hierarchical architecture. They are self-assembled from a dense array of radially-aligned, needle-shaped nanocrystals (nanoneedles), each approximately 25–30 nm in width, creating a well-defined urchin-like morphology. Compared to the typical polyhedral or bulk crystalline morphologies reported in the literature for MIL-53(Al)^37^, this unique architecture indicates that the incorporation of Cd^2+^ ions has fundamentally influenced the coordination geometry and nucleation kinetics during the solvothermal process. The elemental composition and distribution were further investigated by EDS mapping (Fig. 4). The spectrum (Fig. 4a) confirmed the presence of Al, Cd, C, and O, while the mapping (Fig. 4b) revealed their exceptionally uniform distribution throughout the urchin-like structure. The lack of elemental segregation or localized Cd-rich clusters provides strong evidence that the material is a genuine nanostructured bimetallic framework rather than a composite with secondary mineral phases. This structural integration ensures a high density of accessible active sites while maintaining the robustness of the bimetallic framework

#### BET

**Fig. 5:**
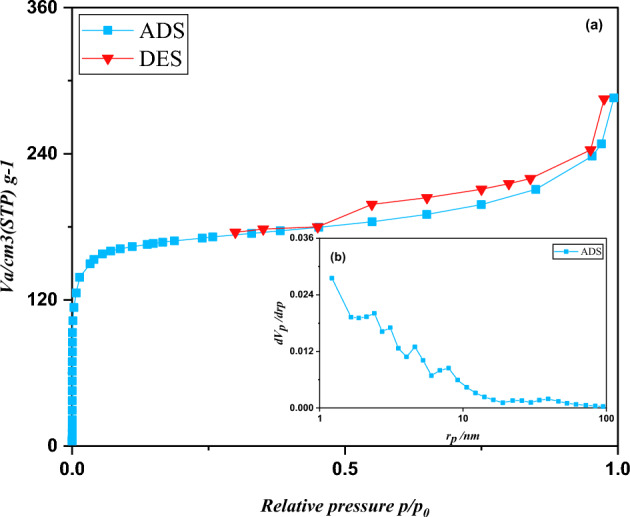
(**a**) N_2_ adsorption − desorption patterns of Al/Cd-BDC MOF (**b**) BJH pore size distribution of Al/Cd-BDC MOF.

The porous nature of the Al/Cd-BDC MOF was investigated via nitrogen adsorption-desorption analysis at 77 K. The material exhibited a Type IV isotherm with a distinct H4-type hysteresis loop (Fig. 5a), which is characteristic of materials containing both micropores and narrow slit-like mesopores^41^. The key textural parameters, summarized in Table 1, confirm the material’s high porosity. The Brunauer-Emmett-Teller (BET) specific surface area was calculated to be 650.8 m^2^ g^–1^. The total pore volume, determined at a relative pressure (p/p₀) of 0.99, was 0.43 cm^3^ g^–1^, with a corresponding average pore diameter of 2.7 nm. Further analysis of the pore size distribution using the Barrett-Joyner-Halenda (BJH) method confirmed these findings. The BJH adsorption cumulative volume of pores was 0.21 cm^3^ g^–1^. More importantly, the pore size distribution plot (Fig. 5b) revealed a very sharp and dominant peak centered at a pore radius of 1.2 nm, corresponding to a diameter of 2.4 nm. The presence of this well-defined, narrow pore distribution, combined with the high surface area, is highly desirable for size-selective adsorption applications and facilitates efficient diffusion of guest molecules to the active sites.

**Table 1:** BET data of Al/Cd-BDC MOF.

Sample	BET surface area (m^2^ g^-1^)	Pore diameter (nm)	Pore volume (cm^3^ g^-1^)	BJH pore diameter (nm)
Al/Cd-BDC MOF	650.8	2.7	0.43	2.4

#### TGA

**Fig. 6:**
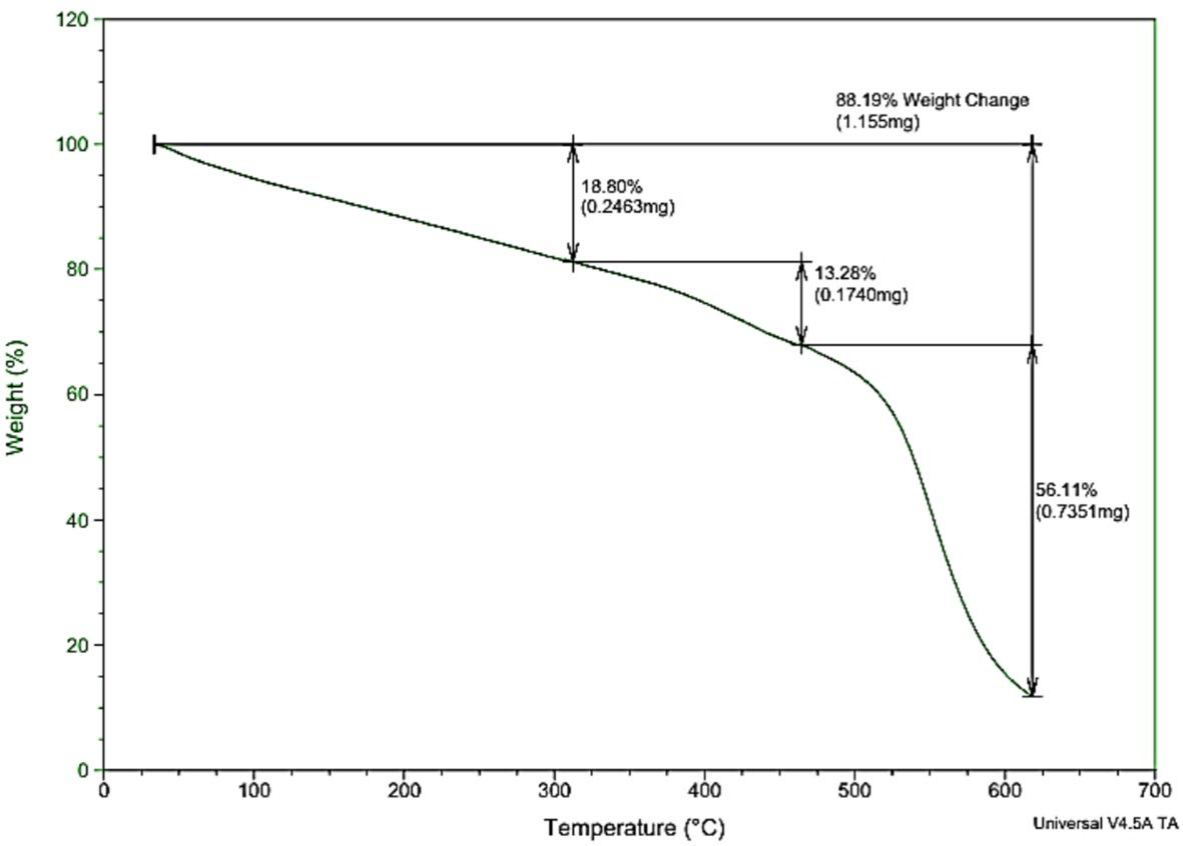
TGA analysis of Al/Cd-BDC MOF.

Thermogravimetric analysis (TGA) was performed to evaluate the thermal stability of the Al/Cd-BDC MOF, as shown in Fig. 6. The thermogram reveals three distinct weight loss stages. The initial weight loss of 18.8% observed below 300°C is ascribed to the removal of guest molecules, such as physisorbed water and residual solvents (DMF and ethanol) from the pores of the framework^42^. Following this, a second, more gradual weight loss of 13.28% occurs between 300°C and 460°C, which can be explained by the sublimation of unreacted H_2_BDC molecules trapped within the framework’s cavities. This interpretation is supported by the known thermal behavior of H_2_BDC, which begins to sublimate above 322°C.^6,43,44^. The main framework decomposition begins at a high onset temperature of approximately 460°C, marked by a major weight loss of 56.11% due to the complete combustion of the BDC linkers and the collapse of the coordination structure^42,45^.The high thermal stability of the Al/Cd-BDC framework, stable up to 460°C, renders it a promising candidate for applications requiring robust materials, such as catalysis and high-temperature adsorption.

### Effects of different parameters on the adsorption of MB dye

#### Effect of contact time

**Fig. 7:**
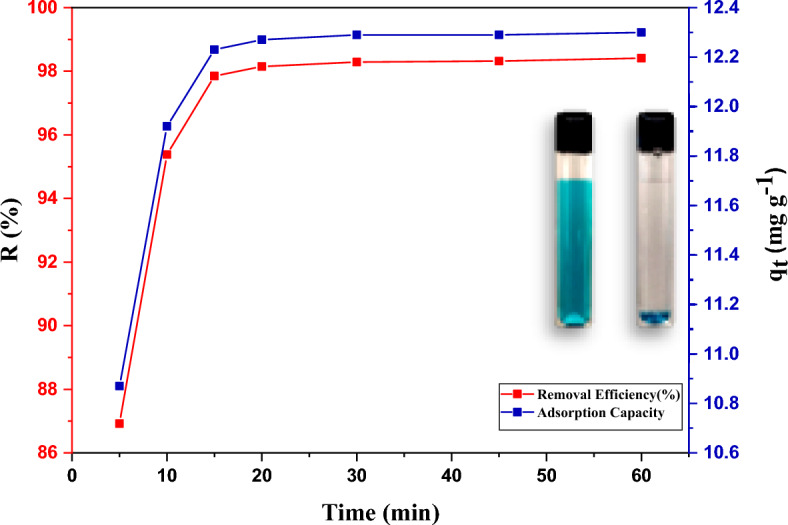
Effect of contact time on the adsorption of MB onto Al/Cd-BDC MOF. Experimental conditions (pH: 7; adsorbent dose: 20 mg; C_o_:10 mg L^−1^; T: 25°C).

The effect of contact time on the adsorption of MB onto the Al/Cd-BDC MOF is presented in Fig. 7. The adsorption process was characterized by two distinct phases: an initial, rapid uptake phase followed by a slower phase as the system approached equilibrium. A substantial amount of MB was adsorbed within the first 15 min, during which the adsorption capacity (q_t_) surged from 10.87 to 12.23 mg g^–1^. This rapid initial adsorption is attributed to the abundance of readily accessible active sites on the MOF’s surface. Subsequently, the rate of uptake decelerated, indicating the gradual saturation of these active sites. An apparent equilibrium was established at approximately 30 min, after which the adsorption capacity remained nearly constant. Based on these results, a contact time of 30 min was selected as the optimal duration for all subsequent experiments to ensure that equilibrium was reached.

**Fig. 8:**
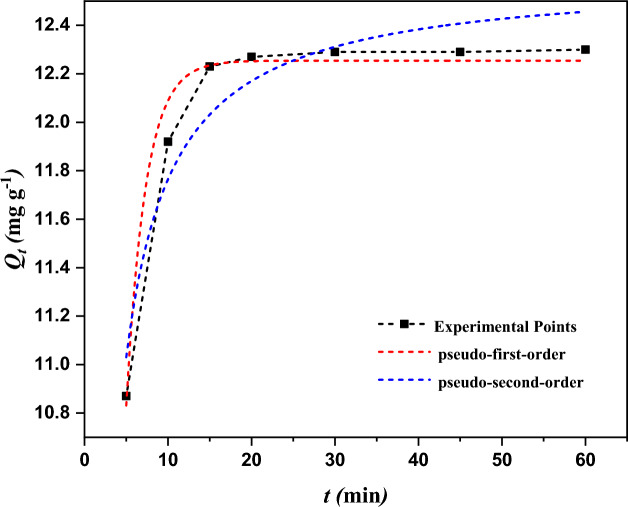
Non-linear regression of pseudo-first-order (PFO) and pseudo-second-order (PSO) kinetic models for MB adsorption onto Al/Cd-BDC (conditions: pH: 7; adsorbent dose: 20 mg; C_o_:10 mg L^-1^; T: 25°C).

To elucidate the adsorption mechanism, the experimental data were analyzed using the non-linear regression of the pseudo-first-order (PFO) and pseudo-second-order (PSO) kinetic models Eq. (3) and (4), respectively^46,47^, as illustrated in Fig. 8.

3 $$Q_{t} = \, Q_{e, \, cal.} \times \, \left( {1 \, - \, e^{ - k1 \times t} } \right)$$

4 $$Q_{t} = \, \left( {Q^{2}_{e, \, cal.} \times K_{2} t} \right) \, / \, (1 + \left( { \, Q_{e, \, cal.} \times \, K_{2} \times t} \right)$$

where Q_e, cal._ (mg g^-1^) is the calculated equilibrium adsorption capacity, K_1_ (min^–1^) and k_2_ (g mg^–1^ min^–1^) are the rate constants for the PFO and PSO models, respectively.

The calculated parameters for both models are summarized in Table 2. The results demonstrate that the PFO model provides a superior fit to the experimental data, yielding a higher correlation coefficient (R^2^ = 0.978) compared to the PSO model (R^2^ = 0.917). Furthermore, the theoretical equilibrium capacity derived from the PFO model (q_e, cal_ = 12.25 mg/g) is in excellent agreement with the experimental value (q_e, exp_ = 12.30 mg g^–1^), whereas the PSO model slightly overestimated this value (q_e, cal_ = 12.6 mg g^–1^). The superior performance of PFO implies that MB adsorption onto Al/Cd-BDC MOF is predominantly governed by physisorption, involving diffusion-controlled mass transport, H-binding, π π interactions, electrostatic attraction, and weak van der Waals

**Table 2:** Kinetic model constants for MB dye adsorption onto Al/Cd-BDC MOF.

Dye	C_o_ (mg L^–1^)	q_e_, exp	Pseudo-first-order			Pseudo-second-order		
			q_e_, cal	K_1_(min^–1^)	R^2^	q_e_, cal (mg g^–1^)	K_2_(g Mg^–1^ Min^–1^)	R^2^
MB	10	12.30	12.25	0.43	0.978	12.6	0.112	0.917

#### Effect of adsorbent dosage

**Fig. 9:**
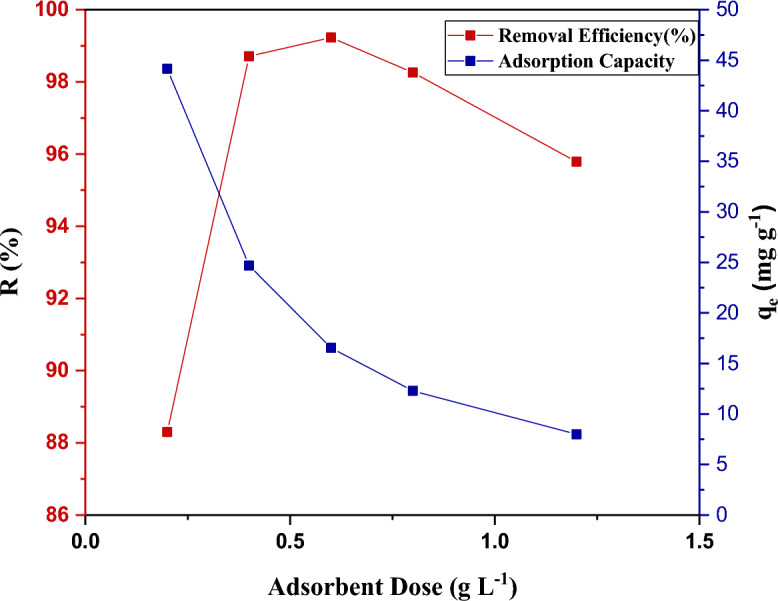
Effect of different doses of Al/Cd-BDC MOF on the removal efficiency of MB. Experimental conditions (pH: 7; t: 30 min; C_o_:10 mg L^-1^; T: 25°C).

The influence of adsorbent dosage on both the removal efficiency and adsorption capacity of MB is depicted in Fig. 9. The removal efficiency exhibited a positive correlation with the adsorbent dosage, increasing from 88.3% to a maximum of 99.23% as the dosage was raised from 0.2 to 0.6 g L^–1^. This improvement is attributed to the greater availability of active adsorption sites and a larger surface area at higher adsorbent concentrations. Conversely, the adsorption capacity (q_e_) showed an inverse trend, decreasing from 44.2 to 8 mg g^–1^ over the same dosage range. This phenomenon occurs because at a fixed initial dye concentration, a higher adsorbent mass leads to a lower ratio of adsorbate molecules to available active sites, thereby reducing the amount of dye adsorbed per unit mass of the adsorbent^48^. Considering that a dosage of 0.6 g L^–1^ (equivalent to 15 mg in 25 mL) achieved nearly complete removal (>99%) without excessive use of the material, it was selected as the optimal dosage for subsequent experiments.

#### Effect of PH

**Fig. 10:**
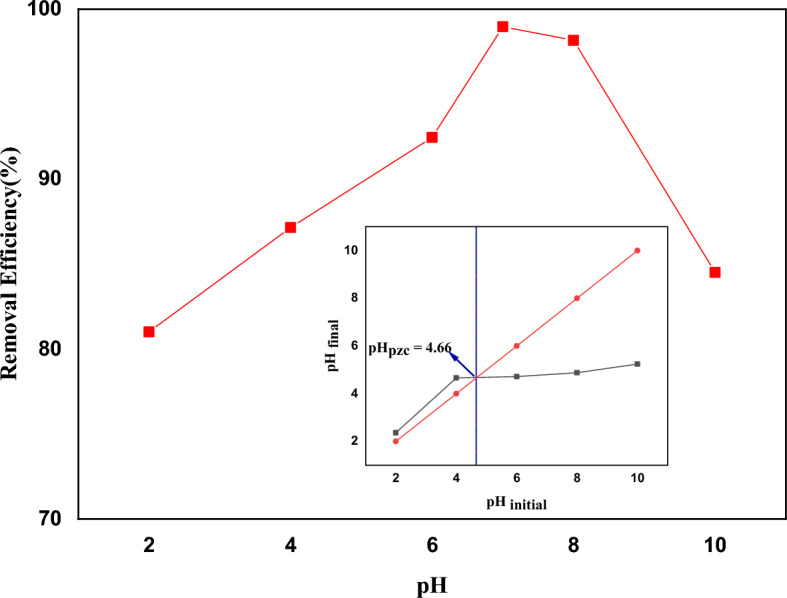
Effect of PH on the MB dye removal efficiency by Al/Cd-BDC MOF (inset: point of zero charge determination). Experimental conditions (adsorbent dose: 0.6 g L^–1^; t: 30 min; C_o_:10 mg L^–1^; T: 25°C).

The initial solution pH is a critical parameter in adsorption processes, as it governs the surface charge of the adsorbent and its electrostatic interaction with the adsorbate. The effect of pH on the removal of the cationic dye Methylene Blue (MB) was investigated, and the results are presented in Fig. 10. The adsorption efficiency was found to be strongly pH-dependent, increasing significantly from 81% at a highly acidic pH of 2 to a maximum of 98.97% at a neutral pH of 7. This behavior can be explained by considering the adsorbent’s point of zero charge (pHpzc), which was determined to be 4.66. At pH values below this point, the MOF surface is protonated and carries a net positive charge, resulting in a strong electrostatic repulsion with the MB^+^ cations that hinders their adsorption. As the pH increases above the pHpzc, the surface becomes progressively deprotonated and negatively charged. This shift minimizes electrostatic repulsion and fosters a strong electrostatic attraction towards the cationic MB molecules. This favorable electrostatic environment, likely complemented by other binding mechanisms such as π-π stacking^49^, results in the enhanced adsorption performance observed as the pH approaches neutrality. Given that the optimal performance was achieved at pH 7, this value was selected as the ideal condition for all subsequent experiments.

#### Effect of initial MB concentration

**Fig. 11:**
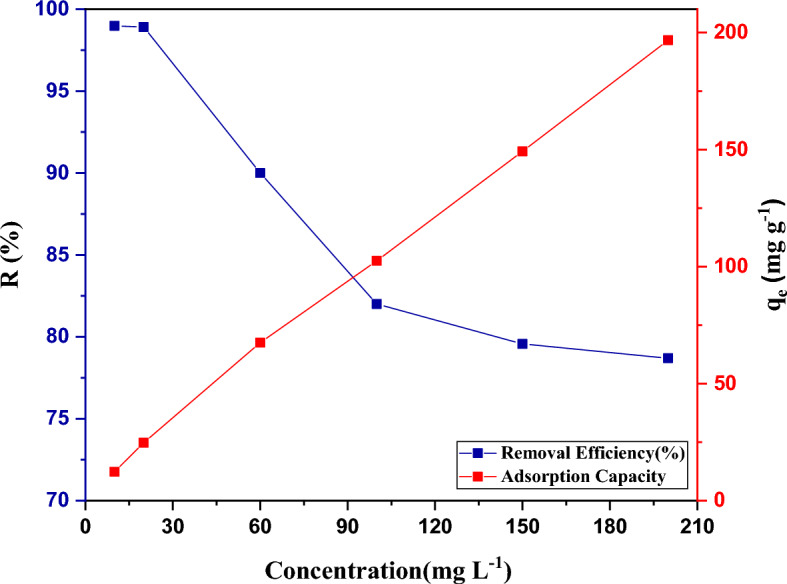
Effect of initial MB concentration on the MB removal efficiency. Experimental conditions (pH: 7; adsorbent dose: 0.6 g L^–1^; t: 30 min; T: 25°C).

The effect of the initial MB concentration (C₀) on both removal efficiency and equilibrium adsorption capacity (q_e_) is presented in Fig. 11. A clear inverse relationship was observed between C₀ and the removal efficiency, which decreased from 98.97% to 78.69% as the concentration increased from 10 to 200 mg L^-1^. This decline is expected, as a fixed adsorbent dosage has a finite number of active sites. At higher concentrations, these sites become saturated, leading to a lower percentage of dye molecules being removed from the solution. In contrast, the adsorption capacity (q_e_) increased significantly with rising initial concentration, from 12.37 mg g^-1^ to 196.73 mg g^-1^. This positive trend is attributed to the enhanced concentration gradient at higher C₀ values. The greater concentration gradient acts as a powerful driving force, overcoming mass transfer resistances and promoting the diffusion of MB molecules from the bulk solution to the surface of the adsorbent.

##### Adsorption isotherms

**Fig. 12:**
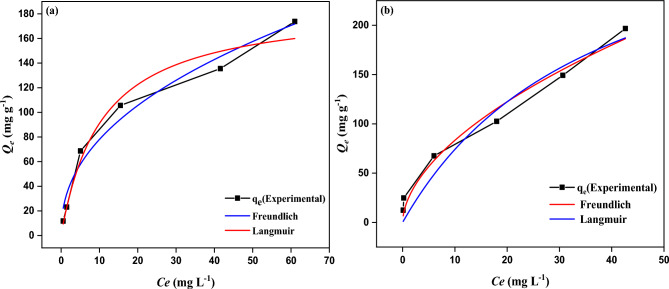
Non-linear fitting of Langmuir and Freundlich isotherm models for the equilibrium adsorption of MB onto Al-BDC (**a**) and Al/Cd-BDC (**b**). Experimental conditions (pH: 7; adsorbent dose: 0.6 g L^-1^; t: 30 min; T: 25°C).

To understand the equilibrium behavior of Methylene Blue (MB) adsorption onto the Al/Cd-BDC framework, the experimental data were fitted to the non-linear Langmuir and Freundlich models (Eqs. (5) and (6) respectively) ^50,51^, as illustrated in (Fig. 12a, b).

5 $$Q_{e} = \, \left( {Q_{m, \, cal.} \times \, K_{L} \times C_{e} } \right) \, / \, \left( {1 + K_{L} \times C_{e} } \right)$$

6 $$Q_{e} = \, K_{F} \times C_{e}^{1/n}$$

where Q_m, cal._ (mg g^–1^) represents the maximum adsorption capacity, K_L_ (L mg^–1^) is the Langmuir constant, K_F_ ((mg g^–1^) (L mg^–1^)^1/n^) and n (dimensionless) indicate adsorption capacity and intensity, respectively

The calculated isotherm parameters and their respective correlation coefficients (R^2^) are summarized in Table 3. The results demonstrate a distinct shift in the adsorption behavior upon the structural modification of the framework. For the monometallic Al-BDC, the data provided a better fit to the Langmuir model (R^2^ = 0.976), suggesting that adsorption primarily occurs on a relatively homogeneous surface via monolayer coverage. In contrast, for the bimetallic Al/Cd-BDC, the Freundlich model yielded a superior fit to the experimental data, as evidenced by a higher correlation coefficient (R^2^ = 0.977) compared to the Langmuir model (R^2^ = 0.949).The Freundlich constant (K_F_), representing the adsorption capacity of the bimetallic MOF, was determined to be 23.18 ((mg g^–1^) (L mg^–1^)^1/n^), while the heterogeneity factor (1/n) was 0.56. The fact that n > 1 (n = 1.78) indicates a favorable adsorption process under the studied conditions. Although the Langmuir model also provided a reasonable fit for the bimetallic system, it yielded a theoretical maximum monolayer capacity (Q_m, cal._) of 352.2 mg g^–1^, which significantly outperforms that of the monometallic Al-BDC (187.9 mg g^–1^). The incorporation of Cd into the Al-BDC framework nearly doubled this capacity, underscoring the synergistic effect of the bimetallic architecture. This structural integration provides a higher density of active sites and a more diverse surface chemistry, making it a superior adsorbent for MB removal. Furthermore, the transition from a Langmuir-dominated fit in the monometallic form to a Freundlich-dominated fit in the bimetallic form confirms that the integration of Cd introduces significant surface heterogeneity. This finding, coupled with the previously established pseudo-first-order (PFO) kinetics, indicates that the adsorption onto the Al/Cd-BDC MOF is a complex process governed by physical interactions across a non-uniform surface architecture with a multilayer distribution.

**Table 3:** Langmuir and Freundlich adsorption parameters for MB adsorption onto Al-BDC and Al/Cd-BDC MOF.

Langmuir model			Freundlich model		
	Al-BDC	Al/Cd-BDC		Al-BDC	Al/Cd-BDC
q_m_ (mg g^-1^)	187.9	352.2	K_F_ (mg g^-1^)(L mg^-1^)^1/n^	28.535	23.18
K_L_ (L mg^-1^)	0.094	0.027	1/n	0.44	0.56
R^2^	0.976	0.949	R^2^	0.972	0.977

#### Effect of temperature and thermodynamic study

**Fig. 13:**
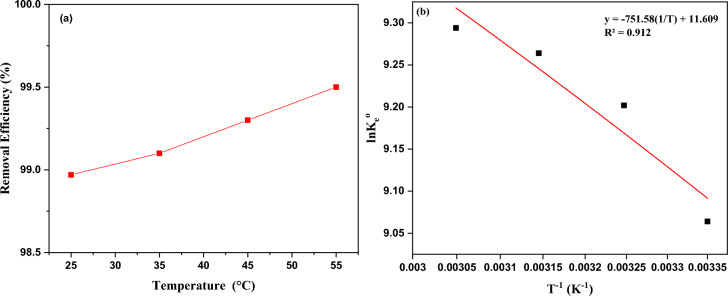
(**a**) Effect of temperature, and (**b**) plots of ln K^°^_e_ versus T^–1^ for MB adsorption onto Al/Cd-BDC MOF.

The influence of temperature on the adsorption of Methylene Blue (MB) was investigated in the range of 25–55°C to determine the process’s thermodynamic feasibility. As shown in (Fig. 13a), the removal efficiency exhibited a clear positive correlation with temperature, indicating that the adsorption process is endothermic in nature. This enhancement can be attributed to the increased kinetic energy of MB molecules, which promotes their diffusion and interaction with the MOF’s active sites^52^. The thermodynamic parameters, including Gibbs free energy (ΔG°), enthalpy (ΔH°), and entropy (ΔS°), were quantified using the dimensionless equilibrium constant (K^°^_e_). K^°^_e_ was derived from the Langmuir constant (K_L_) according to Eq. (7)^53^, and these parameters were calculated via the Van’t Hoff equations (Eq. 8 and 9)^54^, with the corresponding linear plot shown in Fig. 13b.

7 $$K^{^\circ }_{e} = \, \left( {\left[ {Adsorbate} \right]^{0} .MW.K_{L} .1000} \right) \, /\gamma$$

8 $$lnK^{^\circ }_{e} = \, - \Delta H^\circ /RT \, + \, \Delta S^\circ /R$$

9 $$\Delta G = \, - \, RT \, lnK^{^\circ }_{e}$$

where K^°^_e_ represents the thermodynamic equilibrium constant (dimensionless), [absorbate]^0^ is the unitary standard concentration of the adsorbate (1mol L^−1^), MW is the molecular weight of the adsorption (g mol^−1^), K_L_ refers to the Langmuir isotherm constant at different temperatures (L mg^−1^), γ means the co efficient of activity (dimensionless),R is the universal gas constant (Jmol^−1^K^−1^), and T is solution temperature(K).

The calculated thermodynamic values are summarized in Table 4. The negative values of ΔG°, which decreased from −22.46 to −2225.34 kJ mol^−1^ with increasing temperature, confirm that the adsorption process is spontaneous and thermodynamically favorable^55^. The positive enthalpy change (ΔH° = +6.25 kJ mol^–1^) confirms the endothermic behavior. Notably, the magnitude of ΔH° (below 20 kJ mol^-1^) suggests that the adsorption is primarily governed by physical interactions, such as electrostatic attractions and π-π stacking, rather than strong chemical bonding^48,56^. Furthermore, the positive entropy change (ΔS° = +96.52 J mol^–1^·K) reflects an increase in randomness at the solid-liquid interface, likely resulting from the displacement of adsorbed water molecules by the bulkier MB molecules^57^. In conclusion, the thermodynamic analysis reveals that the adsorption of MB onto Al/Cd-BDC is a spontaneous, endothermic, and entropy-driven process. The substantial gain in entropy compensates for the energy requirements (positive ΔH°), making the process highly favorable at ambient and elevated temperatures. These findings underscore the practical potential of the Al/Cd-BDC MOF for efficient wastewater treatment across diverse environmental conditions.

**Table 4:** Thermodynamic parameters of MB adsorption on Al/Cd-BDC MOF.

T (°C)	T (K)	ΔG (kJ mol^−1^)	ΔH (kJ mol^−1^)	ΔS (J mol^−1^ K^−1^)
25	298	–22.46	6.25	96.52
35	308	–23.56		
45	318	–24.49		
55	328	–25.34		

### Comparative performance and synergistic effect

**Fig. 14:**
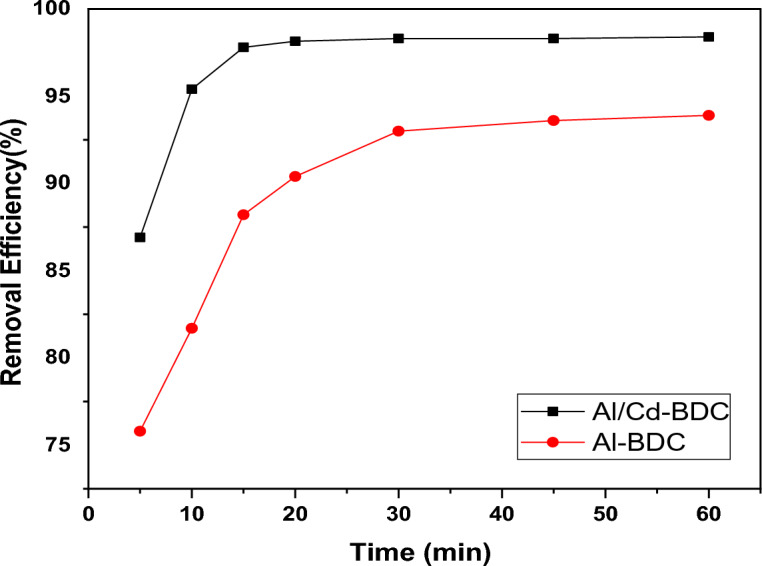
Comparative analysis of MB removal efficiency over time for the bimetallic Al/Cd-BDC and the monometallic Al-BDC. Experimental conditions (pH: 7; adsorbent dose: 0.6 g L^–1^; C_o_: 10 mg L^–1^; T: 25°C).

To validate the superior performance of the bimetallic framework and investigate the underlying synergistic effects, a comparative adsorption study was conducted against the monometallic Al-BDC analogue under identical optimized conditions. The selection of Al-BDC as the host matrix is scientifically motivated by the exceptional hydrothermal stability and permanent porosity characteristic of trivalent metal-carboxylate frameworks. According to the Hard-Soft Acid-Base (HSAB) principle, the Al^3+^ center acts as a hard acid that forms remarkably strong coordination bonds with the carboxylate oxygen atoms (hard base) of the BDC linker, ensuring robust structural integrity in aqueous environments^58^.As illustrated in Fig. 14, the advantages of the bimetallic Al/Cd-BDC are immediately evident; the framework demonstrated rapid kinetics, achieving over 95% removal efficiency within the first 10 minutes and reaching an equilibrium state of approximately 98.5% in just 20 minutes. In contrast, the monometallic Al-BDC exhibited significantly slower uptake, requiring 30 minutes to achieve 93% efficiency. While pure Cd-carboxylate analogues often suffer from limited porosity and reduced hydrolytic stability due to the more labile coordination environments of divalent Cd^2+^ centers (borderline acids) compared to the matched hard-hard Al-O configuration^59^, the strategic incorporation of Cd into the robust Al-matrix in this study creates a powerful synergistic effect. This synergy enhances the framework’s active sites and electronic properties, facilitating a more efficient and rapid adsorption process than the parent monometallic structure. These findings confirm that the bimetallic configuration is essential for maximizing removal performance while maintaining the chemical durability required for practical wastewater remediation.

### Effect of coexisting ions

**Fig. 15:**
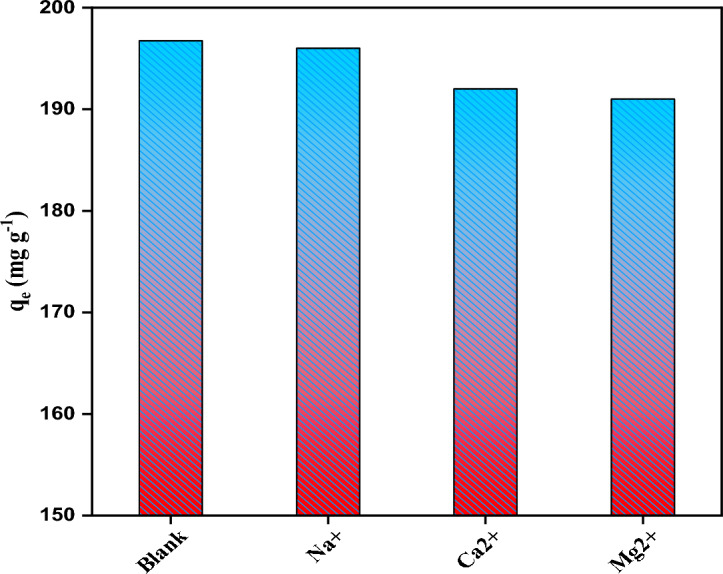
Effect of coexisting inorganic ions (Na⁺, Ca^2^⁺, and Mg^2^⁺) on the adsorption capacity of MB onto Al/Cd-BDC MOF (conditions: pH: 7; adsorbent dose: 0.6 g L^–1^; C₀: 200 mg L^–1^; T: 25 °C; salt concentration: 0.01 M).

The influence of coexisting ions on the adsorption performance was evaluated by introducing common inorganic cations (Na^+^, Ca^2+^, and Mg^2+^) into the dye solution under the optimized experimental conditions. As illustrated in Fig. 15, the adsorption capacity slightly decreased from 196.73 mg g^–1^ in the absence of salts to 196, 192, and 191 mg g^–1^ in the presence of Na^+^, Ca^2+^, and Mg^2+^, respectively. This minor reduction can be attributed to the competition between dissolved cations and dye molecules for the available adsorption sites on the adsorbent surface, as well as to the increase in ionic strength, which may partially weaken the electrostatic interactions between the adsorbent and dye molecules. Notably, divalent cations (Ca^2+^ and Mg^2+^) exhibited a slightly stronger inhibitory effect than monovalent Na^+^ due to their higher charge density^60^. Nevertheless, the adsorbent retained more than 97% of its original adsorption capacity in the presence of these ions, demonstrating a high tolerance toward ionic interference. This behavior suggests that the adsorption mechanism is not governed solely by electrostatic attraction but also involves additional interaction mechanisms. In particular, the aromatic rings present in the organic linker of the framework can promote π–π interactions with the aromatic structure of Methylene Blue molecules, which enhances adsorption stability even in saline environments. Furthermore, hydrogen bonding and pore-filling effects within the porous structure may contribute to the strong affinity between the adsorbent and dye molecules. Consequently, the adsorption performance remains largely unaffected by the presence of competing inorganic ions, highlighting the potential applicability of the material in complex aqueous matrices.

### Sustainability, reusability, and practical relevance

**Fig. 16:**
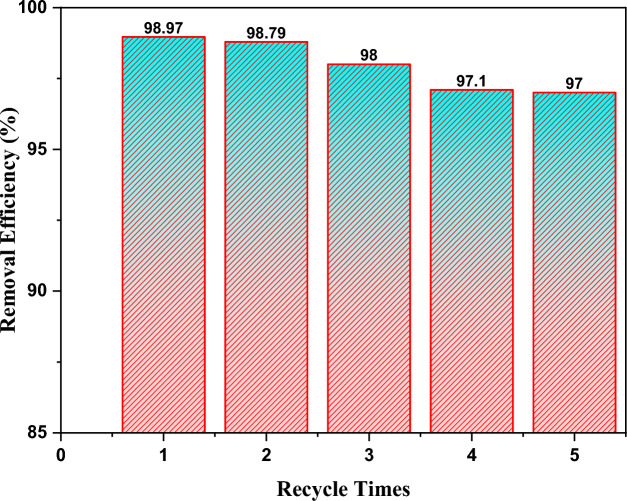
Al/Cd-BDC MOF reusability at (pH: 7; adsorbent dose: 0.6 g L^–1^; t: 30 min; T: 25 °C; C_o_ :10 mg L^–1^).

The practical applicability of the Al/Cd-BDC MOF is firmly established by its inherent sustainability and robust operational stability. Embracing a circular economy paradigm, the synthesis of Al/Cd-BDC MOF leverages terephthalic acid recovered from post-consumer polyethylene terephthalate (PET) waste. This approach substantially lowers raw material expenditures when contrasted with conventional high-purity commercial precursors. Such a ‘waste-to-wealth’ strategy not only confers significant economic benefits but also actively contributes to mitigating plastic pollution. Beyond its sustainable production, the Al/Cd-BDC MOF demonstrates exceptional reusability, retaining over 97% of its initial removal efficiency even after five successive adsorption-desorption cycles (Fig. 16). The regeneration was effectively achieved by washing with ethanol and subsequent drying at 120°C. Post-adsorption characterization further confirms its structural integrity: PXRD analysis (Fig. 1) reveals that the fundamental crystal structure of the Al/Cd-BDC MOF remains well-preserved, while FTIR spectroscopy (Fig. 2) confirms the stability of its main vibrational modes. Collectively, the combination of low-cost precursor sourcing, high cyclic stability, and inherent chemical robustness highlights the material’s promising potential for cost-effective, scalable, and environmentally safe application in industrial wastewater treatment.

### Adsorption capacity comparison

The performance of the synthesized Al/Cd-BDC MOF was benchmarked against various adsorbents reported in the literature for MB removal Table 5[61-69]. With a maximum adsorption capacity (q_m_) of 352.2 mg g^–1^, the Al/Cd-BDC MOF exhibits a highly competitive performance. Notably, its capacity is significantly higher than that of its monometallic Al-BDC analogue, and it surpasses the performance of several other reported frameworks, including the aluminum-based NH_2_-MIL-101(Al), the copper-based CuBDC, and the zirconium-based NH_2_-UiO-66. This superior adsorption capacity strongly suggests a synergistic effect between the Al and Cd metallic centers. This effect, coupled with the high accessibility of active sites afforded by the material’s unique hierarchical morphology, likely accounts for the enhanced MB uptake.

**Table 5:** Comparison of adsorption capacities of various adsorbents for MB removal.

Adsorbent	Experimental conditions	Adsorbate q_m_, (mg g^–1^)	Ref.
CuBDC	pH:6,25°C, m: 1g L^–1^, 20min	41.01	6
Carbon nanotubes	C_o_:20mg L^–1^; pH:7,25°C,90min, m: 15mg	35.4	61
Cu-btc-1	C_o_:10mg L^–1^; pH: 7; T: 40°C; m: 30mg,40min	98.83	49
Zr MOF@Bentonite	C_o_:30mg L^–1^; pH:5; T: 25°C; m: 0.17g, 180min	13.7	62
Fe_3_O_4_@MIL-100(Fe)	C_o_:60 mg L^–1^; pH: 9; T: 45°C; m: 1g	73.80	63
NH_2_- MIL-101(Al)	C_o_:20-40mg L^–1^; pH: 5; T: 30°C; m: 5mg	188	64
MOF-235	C_o_:20-40mg L^–1^; pH: 5.6; T: 25°C; m: 5mg,12h	187	57
MIL101-Cr/PANI/Ag	C_o_:25mg/L; pH: 12; T: 25°C; m: 0.03 g	43.29	65
MOF-199	C_o_:20mg L^–1^; pH: 7.5; T: 25°C; m: 0.2g, 90 min	75.757	66
NH_2_-UiO-66	C_o_:20mg L^–1^; m: 20mg,60 min	96.5	67
Cd-based MOF	C_o_:32mg L^–1^; m: 10mg,24h, at room temperature	79.4	68
MIP-202 Zr-MOF	C_o_:50mg L^–1^; pH: 9; T: 30°C; m: 0.5g L^-1^, 8 min	79.79	69
Al-BDC	C_o_:10mg L^–1^; pH: 7; T: 25°C; m: 15mg,30 min	187.9	This study
Al/Cd-BDC MOF	C_o_:10mg L^–1^; pH: 7; T: 25°C; m: 15mg,30 min	352.2	This study

## Conclusion

This study successfully demonstrated a sustainable "waste-to-wealth" strategy by upcycling post-consumer PET plastic waste into a novel, nanostructured bimetallic Al/Cd-BDC MOF for the efficient remediation of Methylene Blue (MB) contaminated water. The characterization results confirmed that the integration of Al and Cd within the framework created a synergistic effect, resulting in an urchin-like hierarchical morphology and a high BET surface area of 650.8 m^2^ g^–1^, which significantly outperformed the individual monometallic Al-BDC counterpart in terms of adsorption efficiency and structural stability. Under optimized conditions (pH: 7; adsorbent dose: 0.6 g L^–1^; t: 30 min), the bimetallic MOF achieved a remarkable removal efficiency of 98.97% and a high calculated maximum adsorption capacity (Q_m, cal._) of 352.2 mg g^-1^. Thorough investigation using non-linear regression analysis revealed that the adsorption kinetics followed the pseudo-first-order model, while the equilibrium data were best described by the Freundlich isotherm, indicating multilayer adsorption onto a heterogeneous surface. Thermodynamic analysis confirmed the process to be spontaneous and endothermic, with a low enthalpy change (ΔH = +6.07 kJ mol^−1^) that points toward a mechanism dominated by physical interactions, including electrostatic attraction and π-π stacking. Furthermore, the Al/Cd-BDC exhibited exceptional reusability, maintaining over 97% efficiency across five consecutive cycles, showcasing its structural robustness. These findings highlight the Al/Cd-BDC MOF as a superior, eco-friendly, and economically viable adsorbent, offering a dual-action solution for plastic waste valorization and advanced wastewater treatment.

## Data Availability

All data generated or analysed during this study are included in this published article.
